# Automated Photothermal Control of Block Copolymer Self‑Assembly and Metal Oxide Nanostructures Sintering

**DOI:** 10.1002/marc.202600004

**Published:** 2026-03-20

**Authors:** Filip Franciszek Powala, Piotr Szustakiewicz, Przemyslaw Pula, Pawel Wawrzyniec Majewski

**Affiliations:** ^1^ Department of Chemistry University of Warsaw Warsaw Poland

**Keywords:** block copolymer self‐assembly, high‐throughput experiment, machine learning, nanowire sintering, photothermal annealing

## Abstract

We report a microscope‐based photothermal platform that combines structured laser light projection with machine‐learning‐optimized illumination to enable rapid, spatially selective thermal processing of block copolymer (BCP) films and their inorganic replicas. Two neural‐network models were developed: optical‐to‐thermal (O2T), which predicts radial thermal profiles from optical patterns, and thermal‐to‐optical (T2O), which generates the optical pattern required to produce a target thermal field. Using these tools, we achieved near‐top‐hat temperature uniformity over a 1 mm diameter area and accessed temperatures from ∼100°C–350°C for BCP ordering and in excess of 500°C for local sintering/ashing of metal‐oxide nanostructures. The lab‐on‐chip workflow permitted screening of more than 80 process conditions on only a handful of samples and in hours, reducing material and time needs by roughly a factor of 20 vs. conventional thermal annealing. We demonstrate controlled photothermal driving of self‐assembly, rapid local removal of polymer templates, and post‐processing sintering of In_2_O_3_ and Fe_2_O_3_ BCP‐templated nanowires, with correlated changes in carbon residue and nanowire morphology as a function of temperature. The approach enables automated, high‐throughput exploration of optical‐thermal interactions and ordering kinetics, offering a scalable route to accelerate optimization of nanoscale materials and device‐relevant processing.

## Graphical abstract

This study presents a microscope‐based photothermal platform that integrates structured laser light projection with machine‐learning‐optimized illumination. By employing neural‐network models to predict and generate thermal profiles, the platform achieves precise thermal processing of block copolymer films. The approach enables rapid screening of process conditions, reducing material and time requirements significantly compared to conventional methods, and facilitates the exploration of optical‐thermal interactions for material optimization.

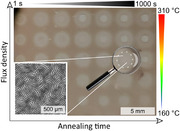

## 1 Introduction

Directed self‐assembly of soft materials, in particular block copolymers (BCP), offers an efficient, scalable route to produce complex nanoscale architectures with low fabrication cost and broad compositional and morphological flexibility [1, 2, 3, 4, 5]. These nanoscale patterns underpin advances in nanolithography [6, 7, 8], optical and photonic devices [9, 10, 11, 12, 13, 14, 15], separation membranes [16, 17, 18, 19, 20], microelectronics hardware fabrication [21, 22, 23, 24, 25], anticounterfeiting [26], and serve as versatile templates for synthesizing a variety of inorganic nanostructures including metals [27, 28, 29], semiconductors [30, 31, 32], and metal oxides [33, 34, 35, 36, 37].

However, reliably translating BCP‐based processes from laboratory demonstrations to robust production remains difficult. Morphological reproducibility is often compromised by batch‐to‐batch variability in starting materials, subtle differences in processing protocols across groups, and sensitivity to environmental and operator‐dependent factors. These issues can mask authentic scientific insights or lead to the dismissal of novel structures as processing artifacts. Standard approaches, for example, thermal or solvent annealing, require exploration of numerous interdependent variables (film thickness, block ratio, annealing temperature and duration, etc.), making optimization laborious, resource‐intensive, and prone to human error.

To address these limitations, automation and even autonomous experimentation have been proposed and are actively being developed to accelerate parameter screening, improve repeatability, and reduce material and labor costs while allowing researchers to concentrate on higher‐level experimental design and interpretation [32, 38, 39, 40, 41] Inspired by these trends, we introduce a lab‐on‐chip strategy for photothermal BCP processing toolbox [42] consolidating many individual annealing experiments on a single substrate. By projecting structured light and using advanced beam shaping to tailor local optical intensity, we precisely control the spatial and temporal temperature landscapes in thin films, enabling spatially selective, programmable photothermal annealing across a substrate. This approach permits simultaneous testing of multiple annealing profiles with minimal material and higher throughput than bulk oven or hot‐plate methods, offers flexible design of space‐ and time‐varying temperature fields and direct coupling between optical patterns and resulting morphologies, and thus facilitates systematic mapping of processing‐structure relationships, improves reproducibility, and helps bridge BCP research and application‐driven fabrication.

## 2 Results and Discussion

### 2.1 Microscope Light Projection System

**FIGURE 1:**
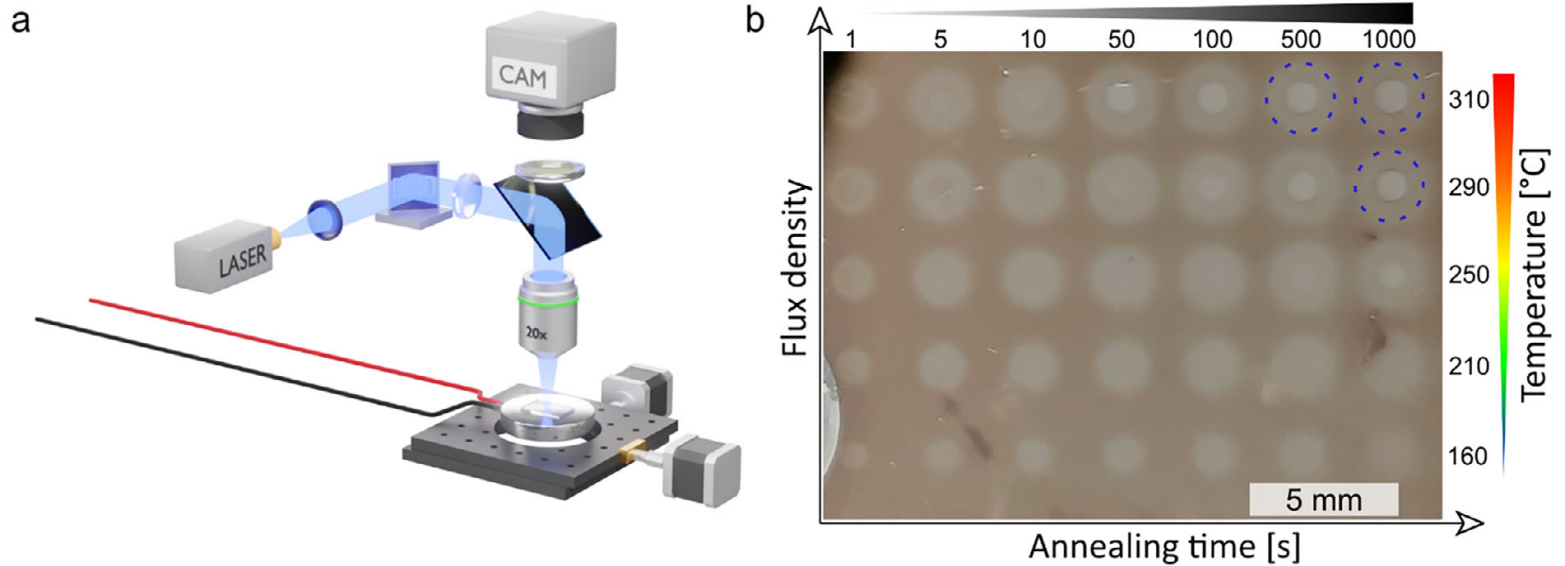
(a) Schematic of the microscopic light‐projection system used for patterned photothermal annealing. (b) Photograph of a single BCP sample on a photothermal glass‐Ge‐Al_2_O_3_ substrate after multiple photothermal annealing runs with varying temperature and exposure time. Dashed circles highlight lighter‐tone, polymer‐dewetted regions produced by prolonged exposure at temperatures >300°C.

The microscope system in Figure 1a uses a 30 W laser diode and a digital micromirror device (DMD) to project structured light via a microscope illumination train (5× objective) onto BCP‐coated glass slides with a thin absorbing layer [43, 44, 45]. It supports photothermal processing of diverse soft materials, from liquid‐crystal films [46] to DNA‐gold nanoparticle superlattices [47]. For this work, the instrument was upgraded with an in situ thermal camera to record temperature profiles and with directed gas flow (inert or O_2_) to enable high‐temperature (200°C–300°C) BCP annealing and laser sintering of BCP‐derived inorganic nanostructures. We used lamellar polystyrene‐*block*‐poly(methyl methacrylate) (L45‐SMMA, *M*_n_ = 45 kg/mol) as a model for ordering‐kinetics studies, and cylindrical polystyrene‐*block*‐poly(2‐vinylpyridine) (*M*_n_ ≈ 259 kg/mol), C259‐S2VP, for synthesizing inorganic metal‐oxide nanowires and subsequent laser sintering.

As shown in Figure 1b, automated illumination routines perform pre‐defined photothermal annealing experiments on thin BCP samples while the stage translates under the microscope. Rather than solve the heat equation for each illumination case [48], we developed a machine‐learning model that predicts the illumination pattern required to produce a desired thermal profile. This data‐driven approach, after initial training, better captures experimental non‐idealities, such as spatial non‐uniformity of the light field and finite thermal resistance at the sample‐substrate interface, that are difficult to model accurately. Precise temperature control is critical for photothermal annealing [48, 49, 50, 51]. For example, a Gaussian profile of laser intensity does not produce a Gaussian temperature distribution, nor does a finite top‐hat beam yield a true top‐hat thermal field [48].

### 2.2 Machine Learning Approach for Predicting Optical Fields

Our method predicts illumination patterns that produce target thermal profiles using machine learning (ML) trained on experimental thermographs. To reduce complexity, we first ran finite‐element simulations of photothermal heating and restricted both optical and thermal fields to radially symmetric cases. Based on these simulations, illumination was parametrized as eight concentric annuli of equal width, each with a constant flux density (Note S1).

We implemented two neural‐network models to link optical patterns and the resulting thermal fields. The optical‐to‐thermal (O2T) model maps measured or simulated optical fluxes (radial profiles from eight concentric annuli) to the resulting Δ*T(r)* thermal profile; the thermal‐to‐optical (T2O) model predicts the optical pattern required to produce a target thermal profile. Training data comprised randomly‐generated optical profiles projected onto the sample and their measured thermal responses, binned radially to form optical‐thermal pairs (see Note S4 and Figure S16a–c). The O2T architecture uses three repeated blocks of two 1D convolutional layers followed by 1D max‐pooling, then a flattened output fed through two dense layers to match the thermal sampling points. We trained O2T with a custom loss that separates mean thermal drift and shape mismatch, which compensates for small environmental drifts between measurements. The T2O model was trained in a closed loop using predictions of the O2T model: T2O proposes an optical profile for a given target temperature, O2T predicts the resulting thermal field, and the loss is computed against the target thermal profile (same loss formulation), avoiding the need for additional thermal measurements (see Figure S16d–f). Both models are robust only for systems similar to the training set (substrates with comparable thermal properties); differences in absorber‐layer thickness can be accommodated by linearly scaling Δ*T* as long as the absorber is much thinner than the substrate.

Similar deep‐learning strategies have been applied to other inverse design problems in optics [52], laser‐aided additive manufacturing [53], and other physical problems, including those governed by nonlinear partial differential equations [54].

**FIGURE 2:**
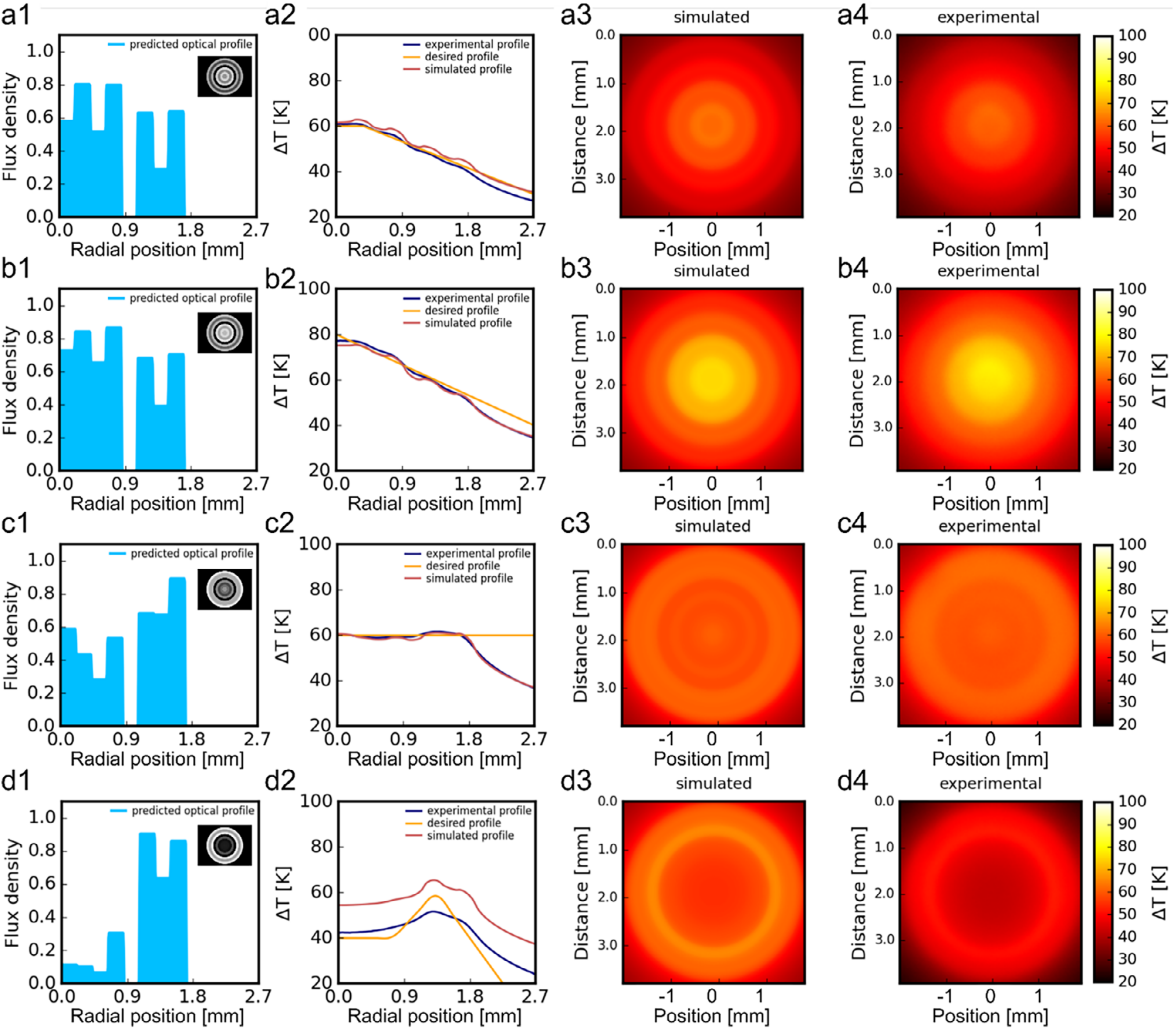
Examples of the machine learning based optical and thermal fields predictions (a–d). Using the T2O model, radially‐symmetric illumination patterns (panels a1, b1, c1, and d1 show their radial flux density profiles) are predicted by the trained neural network based on the requested thermal profiles (orange curves in panels a2, b2, c2, d2). The accuracy of the predictions can be verified by finite‐element numerical simulations and experimental thermography (a3, b3, c3, d3, and a4, b4, c4, d4, respectively). The 1D temperature profiles obtained by simulations and thermography are shown in panels a2, b2, c2, and d2 as brown and dark‐blue curves, respectively.

Figure 2 shows examples produced by the T2O model: constant‐slope radial gradients (Figure 2a,b), quasi top‐hat profiles (Figure 2c), and non‐monotonic “crater‐like” profiles (Figure 2d). The ML approach accurately predicts feasible optical patterns for any physically realizable target thermal field. A given optical profile produces a family of homologous thermal fields by scaling the incident power, Δ*T* ∝ *k*·*P*_0_ (Δ*T* is the temperature rise above base; *P*_0_ is the projected flux density). The inverse problem is generally degenerate: multiple optical profiles can produce similar thermal fields, owing to the thermal camera resolution (±1°C) and lateral heat diffusion, which smooths fine features.

Some target profiles remain challenging or impossible. A perfectly flat thermal profile across the simulated region conflicts with boundary heat dissipation and cannot be generated or included in training. Very steep positive gradients in the inner region are difficult because increasing flux in outer annuli raises the entire profile via lateral conduction. We further focused primarily on top‐hat thermal profiles for BCP annealing and ashing. Despite limited training data on extreme cases (e.g., thermal wells or sharp internal steps), the model can still predict many such profiles, which may be useful for other photothermal soft‐matter experiments [46] and laser‐aided materials processing [53, 55].

### 2.3 Grain Coarsening Kinetics in BCP Thin Films

We used top‐hat thermal profiles (Figure S3) to perform photothermal grain‐coarsening experiments on L45‐SMMA thin films of varying thickness. Top‐hat profiles provide a less arbitrary determination of the local temperature (controlled here to ±5°C) and can be directly related to the most popular thermal annealing methods (e.g., vacuum oven or hot plate) where in‐plane spatial temperature gradients are minimal and, unlike in zone‐heating, do not affect self‐assembly kinetics [56, 57]. The maximum profile temperature was limited to 310°C because higher temperatures produced almost instantaneous film dewetting (Figures S7 and S8).

We quantify BCP self‐assembly kinetics by the grain size *ξ*, obtained from the extent of the autocorrelation of azimuthal domain orientation in SEM images [58]. The growth follows an empirical power‐law model [59]: *ξ* = *A t* ^α^ where *A* is a pre‐exponential (temperature‐dependent) factor, and *α* is the kinetic growth exponent. In principle, *α* is temperature‐independent. Thanks to a uniform temperature profile across the patterned zone, the BCP grain size is highly homogeneous, with a standard deviation of *ξ* of 5 % over the central 80 % of the illuminated region (Figure S5).

**FIGURE 3:**
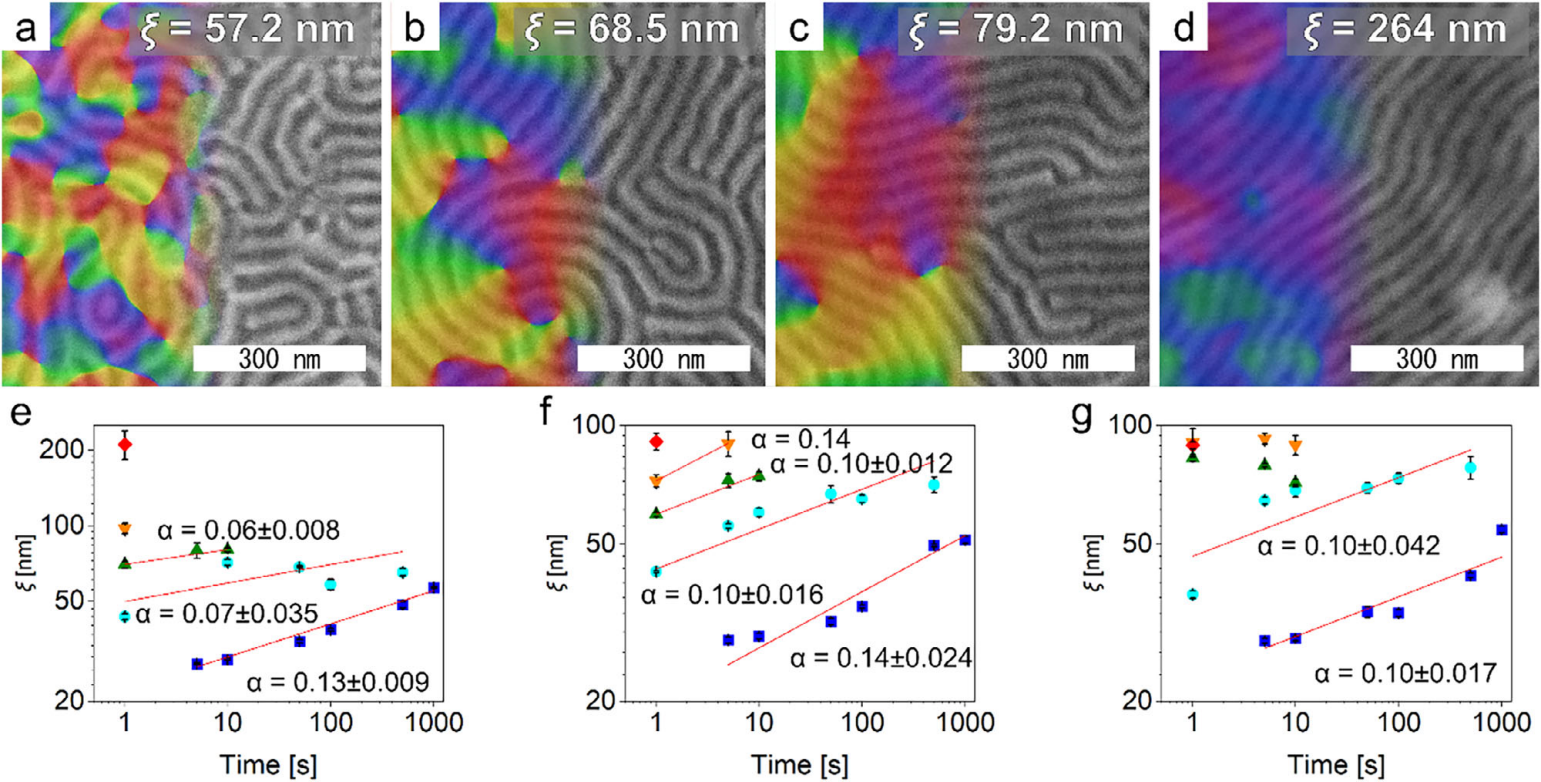
SEM images with false‐color grain‐orientation maps of L45‐SMMA films (thickness 23 nm) processed at: (a) 160°C, 1000 s; (b) 210°C, 50 s; (c) 250°C, 10 s; (d) 310°C for 1 s. Scale bar is 300 nm. (e–g) Correlation length ξ vs. illumination time for films of thickness 21 nm (e), 25 nm (f), and 28 nm (g). Solid lines are the best fits to the kinetic power‐law model. Color code: 160°C (blue), 210°C (cyan), 250°C (olive), 290°C (orange), 310°C (red).

Experiments were performed on L45‐SMMA films of thickness 28, 25, and 23 nm, roughly commensurate with the BCP period *L*_0_ = 32 nm, and which form vertically oriented lamellae on substrates coated with a random PS‐*r*‐PMMA brush. For each thickness, we tested a matrix of conditions: five temperatures between 160°C and 310°C and anneal times from 1 to 1000 s. As shown in Figure 3a–d, temperature has the strongest effect on ordering kinetics. Notably, in 23 nm films, the grain size after only 1 s at the highest temperatures can exceed 10·*L*_0_. Grain growth is not sustained at these high temperatures: at prolonged anneals (e.g., 290°C and above), the BCP thermally degrades, and the film dewets the substrate (dewetting is faster and more pronounced for thinner films).

The apparent initial grain size (reflected in *A*) is largest for the thinnest films and, as previously reported, increases with the annealing temperature [60]. Conversely, the kinetic exponent *α* is larger for thicker films: for example, at 210°C, *α* = 0.07 for the 21 nm film, vs. 0.10 for the 25 and 28 nm films. Regions with visible thermal degradation were not included in grain‐coarsening kinetic plots shown in Figure 3e–g. Examples of such regions are demonstrated in Figures S7 and S8. We note that peak temperature predominantly controls dewetting of L45‑SMMA: at high T, the films develop small, uniformly distributed holes consistent with homogeneous nucleation, as reported by Gianotti for a similar PS‑*b*‑PMMA lamellar system [61].

Reported *α* values for thermally‐annealed BCP vertical lamellae or horizontal cylinders (i.e., stripe patterns) typically range from ∼0.02–0.3 [56, 59, 60, 62, 63]. Higher defectivity and lower grain‐coarsening exponents have been reported for thinner films [64] except for the films where island formation and near‐edge BCP ordering are observed [45, 65]. Our results for samples annealed at 210°C and 250°C are consistent with this trend. Nevertheless, we acknowledge that the narrow range of film‐thickness with stable vertical lamellae morphology of L45‐SMMA had limited broader screening.

To compare with hot‐plate annealing, we prepared matching L45‐SMMA films and annealed them at fixed temperatures for defined times; SEM kinetics are in Figure S6. Laser annealing outperforms hot‐plate annealing for short, high‐temperature transients (5–10 s), yielding larger grains and higher pre‐exponential factors *A*, because laser heating delivers very short thermal pulses while samples on a hot plate need ≈ 15 s to reach 90 % of the target Δ*T* (simulated and measured). Dewetting occurs in both methods but appears much later for hot‐plate annealing, reflecting the time lag between the hot‐block setpoint (verified with a calibrated TC probe) and the offset between the film surface and hot‐plate temperature. Slower surface heating and increased oxidative degradation in hot‐plate annealing likely cause the near‐arrested grain growth observed at nominal block temperatures of 160°C and 210°C.

Finally, we note that each thickness series used a single 25 × 25 mm^2^ sample; the full experiment spanning five temperatures and six time points can be completed in roughly 2 h, including film deposition, which is a significant productivity increase as compared to experiments on individual samples annealed in a vacuum oven.

### 2.4 Laser Heating in Fabrication of BCP‐Derived Metal Oxide Nanowires

We used high‐molecular‐weight C259‐S2VP to template inorganic metal‐oxide nanowires and to subject them to subsequent laser sintering. The BCP was loaded with Fe(acac)_3_ or In(acac)_3_ precursors, which co‐assembled into precursor‐filled cylindrical P2VP domains during casting on the photothermal substrates [35, 37]. To convert these precursor‐hosting domains into inorganic nanowire (NW) replicas, the organic template must be fully removed. Common approaches, including oxygen plasma ashing [27] or UV/ozone treatment [66], are effective but limited by plasma penetration depth and can leave embedded organic residues. High‐temperature calcination (400–1000°C) allows complete polymer removal and high‐purity refractory metal [28] or semiconductor [67] nanostructured frameworks, but generally requires careful control because excessive annealing can sinter and collapse fine features, disrupting their connectivity even as it improves crystallinity.

**FIGURE 4:**
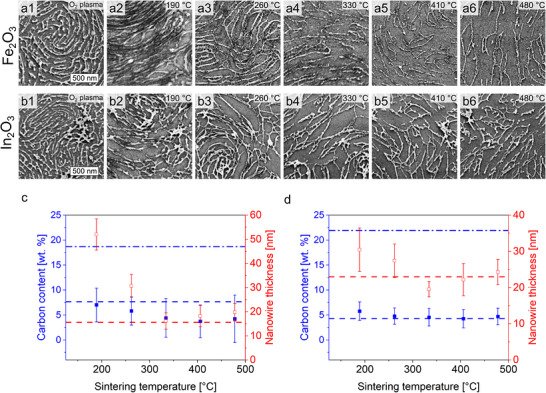
SEM images of Fe_2_O_3_ (a) and In_2_O_3_ (b) nanowires produced through BCP template ashing in oxygen plasma for 20 min (a1 and b1) and photothermally heated to specified temperatures (a2–a6, b2–b6) for 300 s in an oxygen atmosphere using a laser setup. Residual carbon content measured by EDS, and the nanowire diameters for the laser‐sintered Fe_2_O_3_ (c) and In_2_O_3_ (d). Solid blue squares indicate carbon content in weight percent. The dash‐dot blue lines represent the initial carbon content in the precursor‐infused BCP matrix, while the dashed blue lines illustrate the carbon content observed in samples ashed for 10 min in oxygen plasma. Open red squares denote the nanowire thickness after photothermal sintering, and the red dashed lines mark the diameters of the nanowires following oxygen plasma processing. The error bars in carbon content represent the standard deviation based on at least five individual measurements, while the error bars for nanowire diameter indicate the standard deviation from 50 individual measurements. Each scale bar corresponds to 500 nm.

In this study, we used laser annealing to both ash the BCP template and sinter the oxide NWs in a single step. Laser processing shortens the workflow, eliminates the need for oxygen plasma, and permits local material annealing or maskless surface patterning [67, 68]. Switching to a 10× microscope objective increased the photon flux density and raised peak processing temperatures by roughly a factor of two. We processed 100 nm BCP films for 3 min at conditions producing estimated peak temperatures from ∼190°C to ∼480°C in an oxygen atmosphere – well‐below the bulk melting points of Fe_2_O_3_ (1539°C) (Figure 4a2–a6) and In_2_O_3_ (1910°C) (Figure 4b2–b6).

Laser ashing in the presence of oxygen produced carbon‐residue levels and NW thicknesses comparable to oxygen plasma treatment (Figure 4a1,4b1), as measured by energy‐dispersive X‐ray spectroscopy (EDS) and SEM, but with much shorter processing times (Figure 4c,d). Even relatively low laser‐processing temperatures removed most organic material; effective inorganic sintering (wire thickness approaching plasma‐processed levels) was achieved by ∼330°C, with little further thickness change at higher temperatures. Nonetheless, at the highest processing temperatures (> 410°C), partial breakup of continuous wires into a bead‐of‐pearls morphology reduced NW continuity, and NW detachment from the substrate was observed (Figure 4a6,b6). We also observed that the composition of the gaseous atmosphere, that is, oxygen vs. air, during calcination generally enhances laser ashing by reducing the carbon content in the processed nanomaterials (Figures S10 and S11 show in‐air sintering results). Improved NW crystallinity after high‐temperature annealing was previously confirmed for thicker BCP‐templated Fe_2_O_3_ [35] and In_2_O_3_ [37] replicas using IR‐laser rapid thermal annealing. Here, the monolayer‐thin nanowire volume was too small for reliable analysis by laboratory X‐ray diffraction.

Finally, because laser ashing/sintering fixes the NWs to the substrate, locally treated regions are no longer removable by common solvents (e.g., toluene, THF). This property enables direct, localized patterning of various oxide NWs on a single substrate.

## 3 Conclusions

Precisely controlled thermal treatment strongly determines the final properties of nanostructures derived from BCP templates and therefore controls their functional performance in catalysis, sensing, and electronic applications. Here we show that photothermal laser processing can both drive BCP self‐assembly and perform local high‐temperature ashing and sintering of the resulting inorganic replicas, enabling rapid exploration of ordering kinetics and direct optimization of nanowire morphology and connectivity. Integrating machine‐learning in guided control of spatially modulated illumination and dynamic laser protocols promises to elevate this approach by predicting and stabilizing complex light‐and‐thermal‐field scenarios that are difficult to treat with conventional methods. Finally, the microscopic photothermal platform and a lab‐on‐chip workflow facilitate faster, automated screening and characterization, accelerating device development and opening pathways to new materials discoveries and industrially‐relevant optimizations.

## 4 Experimental Section

### 4.1 Polymeric Materials

A lamellae‐forming polystyrene‐*block*‐poly(methyl methacrylate) with a molecular weight of *M*_n_^PS^ = 23 kg/mol, *M*_n_^PMMA^ = 22 kg/mol, and a dispersity (*Đ*) of 1.10 (Polymer Source), hereafter referred to as L45‐SMMA, was dissolved in toluene (GPC grade, Carl Roth) to obtain a 1 % w/w solution. Additionally, a random polystyrene‐*r*‐poly(methyl methacrylate) with *M*_n_ = 48 kg/mol, *M*_w_ = 72 kg/mol, and a *Đ* = 1.5 (54 mol. % PS, Polymer Source) was dissolved in propylene glycol methyl ether acetate (PGMEA) to form a 0.5 % w/w solution.

For the In‐ and Fe‐infused BCP films, we utilized a cylinder‐forming polystyrene‐*block*‐poly(2‐vinylpyridine) with *M*_n_^PS^ = 182 kg/mol, *M*_n_^P2VP^ = 77 kg/mol, and a *Đ* = 1.09 (Polymer Source), referred to as C259‐S2VP. The BCP was dissolved in a mixture containing 80 wt. % toluene (GPC grade, Carl Roth) and 3,4,5‐trimethoxytoluene (97 %, TCI Chemicals, TMOT) to form a 1.5 % wt./wt. BCP solution. The C259‐S2VP stock solution was then mixed with respective 3 wt. % solutions of In(acac)_3_ or Fe(acac)_3_ to yield 1 wt. % BCP solutions with metal‐to‐vinyl pyridine molar ratios of Fe:VP = 1:2 and In:VP = 3:8, which were subsequently used for thin film preparation according to the protocols reported by Pula et al. [35, 37].

### 4.2 Fabrication of Photothermal Substrates and BCP Films Deposition

Photothermal substrates were fabricated using standard microscopic glass slides (75 × 25 × 1 mm^3^) coated with a 100 nm‐thick layer of germanium (N‐type, 99.999 %, Kurt J. Lesker) via thermal evaporation (NanoPVD T15A, Moorfield Nanotechnology). A protective 20 nm‐thick alumina layer was subsequently added using atomic layer deposition (BeneQ TFS 200). The rationale for the selection of the substrate and absorber, along with a discussion of alternative substrate materials, is provided in Note S1.

Before spin‐coating, the photothermal substrates were treated with oxygen plasma (PE‐25, Plasma Etch, 200 mTorr O_2_, 100 W RF power, 120 s) to clean and activate the surfaces. Each spin‐coating was conducted at room temperature (23°C) in a spin‐coater (SPIN 150i, SPS) under a dry air flow of approximately 2 L/min. A 95 µL aliquot of the random polymer solution was deposited onto a stationary 25 × 25 mm^2^ substrate, followed by 60 s of spinning at 3000 rpm. The coated substrates were then soft‐baked on a hot plate for 5 min at 220°C, cooled down, and returned to the spin‐coater. They were rinsed with toluene and spun again at 3000 rpm for 60 s. Next, a brush‐functionalized substrate was spin‐coated with L45‐SMMA BCP solution to achieve film thicknesses of 21–28 nm (Table S1 and Figure S4).

For the In‐ and Fe‐infused BCP thin films, spin‐casting of C259‐S2VP with metal precursors was performed at 2000 rpm for 120 s to create a ∼100 nm thick layer utilizing the direct casting and ordering procedure described elsewhere [35]. To expedite non‐volatile solvent (3,4,5‐trimethoxytoluene) evaporation and enhance film ordering, the sample was heated on a hot plate at 60°C for 5 min.

Polymer film thickness was verified by X‐ray reflectometry (D‐8 Discover, Bruker, parallel‐beam geometry), configured with a 0.1 mm lamp and detector slits. The reflectivity curves were recorded over a *2θ* range of 0.1°–5° with a step size of 0.01° and fitted using Leptos software.

### 4.3 Photothermal Experiments

A 30 W, 455 nm laser source (Opt Lasers) was connected via optical fiber to a light collimator (*f* = 50 mm, air‐spaced achromatic doublet) used as a source of illumination in the microscope projection system described in detail elsewhere [46, 47]. This optical system allows for arbitrary shaping and control of light flux intensity within the microscope's field of view (Nikon EPI‑Plan 5× objective) using a digital micromirror device (DMD, 0.45″, 1024 × 768 pixels, Texas Instruments). The sample was placed on a motorized stage (Thorlabs M30XY/M) equipped with an ITO (for in‐line micro‐thermographic measurements) or a copper‐plate heater (for photothermal annealing), enabling precise temperature control of the substrates via an external controller (Thorlabs TC300); silicone vacuum grease facilitated thermal contact between the sample and the heaters. To assess thermal fields induced by sample illumination, a thermographic camera (Optris, Xi400) was used, paired with a custom‐designed germanium macro‐objective. The setup allows for thermal measurements in both epi‐observation (at a 45° angle to the laser axis) and transmission geometry. However, due to reduced image distortion in transmission mode, most thermal measurements were conducted in the transmission geometry on ITO substrates. These measurements, collected under the steady‐state conditions, provided the basis for simulations and machine‐learning experiments. To avoid BCP oxidation during laser‐induced BCP ordering experiments, a gentle stream of nitrogen (1 L/min) was directed at the sample surface coaxial with the laser illumination axis.

### 4.4 Thermal Simulations

Thermal simulations were conducted using COMSOL Multiphysics 5.2a software. The simulated geometry featured a constant‐temperature base plate with a mounted photothermal substrate. Light absorption in the Ge‐Al_2_O_3_‐BCP stack was modeled using experimentally recorded absorption data for 450 nm light, measured at normal incidence with a spectral reflectometer (F‐20, Filmetrics). In the geometric model, the thicknesses of the germanium, alumina, and BCP layers were neglected, treating heating as a surface heat source and modeling laser illumination as a boundary heat source. To replicate experimental conditions, the base temperature was set to 100°C. Thermal contact between the sample and the base was defined using the Cooper‐Mikic‐Yovanovich correlation, with a gap conductance (*h*_g_) of 100 W/(m^2^·K) [51]. All outer surfaces, except for the bottom base, were treated as sources of heat flux, with a heat transfer coefficient (*h*) of 20 W/(m^2^·K) and an ambient temperature of 25°C. Patterns were applied according to the image on the sample's upper surface. Calibration of power levels (Figures S1 and S2) was performed between the 8‐bit grayscale images sent to the DMD and the actual optical flux intensity measured at the sample plane using an optical power meter (PM100D console with S415C head, Thorlabs). An auxiliary coefficient (*k*) was introduced to scale the incident flux density to the absorbed light power, accounting for light losses primarily due to reflection. Iterative simulations were conducted to achieve agreement with experimental data. A list of adjusted parameters, including *h*_g_, and *h*, along with other thermal simulation parameters, is provided in Note S3. Simulations also validated thermal profiles produced with high‑magnification microscope objectives, i.e., 20×, resolving fine thermal‐field features that lie below the spatial resolution of our thermal camera.

### 4.5 Optimization of Optical Profiles by Machine‐Learning Approach

The optical profiles (OPs) used here were radially symmetric, and the resulting thermal profiles (TPs) shared the same symmetry. We generated 543 random OPs; for each OP, the corresponding TP was recorded with a thermographic camera after 10 s of pattern illumination, at the thermal steady‐state. The illumination was repeated at least three times over different positions on the sample to ensure the reproducibility of the resultant thermal fields.

From the paired OP‐TP dataset, we developed two Keras/TensorFlow models in Python. The first model, optical‐to‐thermal (O2T), was trained to predict a TP from a given OP. The second model, thermal‐to‐optical (T2O), performed the inverse task: given a target TP, it predicted an OP (see the diagram in Note S4). Training the T2O model used randomly generated TPs as inputs; the OPs predicted by T2O were passed through the trained O2T model to produce simulated TPs, which were compared to the targets to compute the loss. Both models were trained on an NVIDIA RTX 3070 GPU under Windows Subsystem for Linux. To reduce experimental 2D thermal fields to 1D profiles, we computed radial averages: for OPs, the grayscale optical flux (normalized between 0 and 1); for TPs, the temperature increase Δ*T* above base temperature (100°C), each averaged as a function of radial distance from the center.

### 4.6 BCP Ordering Kinetics and Laser‐Ashing of Polymer Matrix

For the ordering‐kinetics measurements, L45‐SMMA films of various thicknesses were photothermally annealed using optical profiles that produced a top‐hat temperature profile with ±5°C uniformity around the target temperature within the central 1 mm of the heated region. Samples were translated automatically and exposed for prescribed durations under a Python control script. Individual photothermal fields were spaced 4 mm apart to avoid overlap. Annealing used a 100°C base temperature (resistive heating) and an N_2_ gas shroud to prevent oxidation. For laser removal of BCP templates from In(acac)_3_‐ or Fe(acac)_3_‐infused samples, a gentle stream of oxygen or dry air was delivered to the heated region during irradiation.

### 4.7 Scanning Electron Microscopy and Image Analysis

To enhance contrast during SEM imaging of L45‐SMMA films, we selectively converted PMMA into an Al_2_O_3_ replica through three cycles of sequential exposure to trimethylaluminum and water vapor at 85°C in a custom‐built sequential infiltration synthesis reactor operating at a base pressure of approximately 2 Torr. Organic residues were removed using oxygen plasma etching (PE‐25, Plasma Etch; 20 mTorr O_2_, 100 W RF power, 10 min). SEM images were acquired with a Zeiss Merlin FEG‐SEM, utilizing an in‐lens secondary electron detector at an operating voltage of 2 keV. Grain size was determined from the SEM images as the characteristic decay length of the autocorrelation function of the domain orientation *g(r)*. This was achieved by fitting *g(r)* to an exponential function *e^−r/ξ^* using Python routines from the SciAnalysis package [58]. The *ξ* values represent the average of three measurements for each annealing condition. Error bars indicate expanded uncertainty, with B‐type uncertainty derived directly from the SciAnalysis package and type A uncertainty calculated as the standard deviation of the mean. For the assessment of In_2_O_3_ and Fe_2_O_3_ thickness, we used ImageJ software. Additionally, a quantitative EDS analysis was performed to evaluate the carbon content in samples subjected to laser annealing. This analysis was conducted using a Zeiss Merlin‐2 SEM equipped with a Bruker X‐Flash Detector 5010 (125 eV resolution) and the Quantax system at 15 kV. Each sample was tested in 3 different areas.

## Author Contributions

Filip F. Powala: research conceptualization (supporting), methodology development, investigation (lead), drafting manuscript, data curation, and visualization. Piotr Szustakiewicz: investigation (lead—ML methodology), manuscript writing (supporting), visualization. Przemyslaw Pula: investigation (supporting—BCP templated synthesis of inorganic nanostructures), data analysis, manuscript writing (supporting). Pawel W. Majewski: research conceptualization, methodology development, data analysis, manuscript writing, funding acquisition, and project supervision.

## Funding

The authors acknowledge financial support from the National Science Centre of Poland (NCN) program Sonata Bis 10, grant number UMO‐2020/38/E/ST5/00328 for light‐directed self‐assembly of complex soft materials.

## Conflicts of Interest

The authors declare no conflict of interest.

## Supporting information




**Supporting File**: marc70261‐sup‐0001‐SuppMat.pdf.

## Data Availability

The data that support the findings of this study are available from the corresponding author upon reasonable request.
