# Supplementary material for: Automated Photothermal Control of Block Copolymer Self‑Assembly and Metal Oxide Nanostructures Sintering
Source: Macromol Rapid Commun. 2026 Mar 20;47(13):e00004. doi: 10.1002/marc.202600004 (PMC13331546; doi:10.1002/marc.202600004)
Supplement: Supplementary file 1 — Supporting File: marc70261‐sup‐0001‐SuppMat.pdf. [file MARC-47-e00004-s001.pdf]

# Supplementary Information for:

## Automated Photothermal Control of Block Copolymer Self-Assembly and Metal Oxide Nanostructures Sintering

*Filip Powala[1], Piotr Szustakiewicz[1], Przemyslaw Pula[1], Pawel W. Majewski[1]**

**Email: pmajewski@chem.uw.edu.pl*

[1]Department of Chemistry, University of Warsaw, Warsaw, 02093, Poland

**Contains:**

**Note 1** – Calibration and Setup of Photothermal Experiments

**1.1** – Calibration of Incident Laser Power

**1.2** – Substrate and Coating Considerations

**Note 2** – Block Copolymer Thin Film Preparation and Laser Annealing

**2.1** – BCP Film Thickness Verification

**2.2** – Uniformity of BCP Grain Size within Illuminated Zone

**2.3** – BCP Ordering Kinetics in Control Hot-Plate Annealing

**2.4** – Thermal Degradation and Dewetting of BCP Films

**2.5** – BCP conversion to $Al_2O_3$, BCP ashing and Metal Oxide Nanowire Sintering

**Note 3** – Thermography and Numerical Simulations of Photothermal Heating

**3.1** – Thermography

**3.2** – Numerical Simulations

**Note 4** – Machine Learning in O2T and T2O Models

## Note 1 – Calibration and Setup of Photothermal Experiments

**1.1** – Calibration of Incident Laser Power

Laser power density (flux) was measured with a thermal power meter placed in the sample plane. The illuminated area was taken as the physical diameter of a rectangular or circular pattern displayed on the DMD, demagnified by the microscope objective; demagnification was independently verified using a microscope calibration target. Calibration curves in **Figures S1** and **S2** were obtained with a 5× objective. Beam power at the DMD was adjusted by changing the laser duty cycle in pulse-width-modulation mode at 2 kHz.

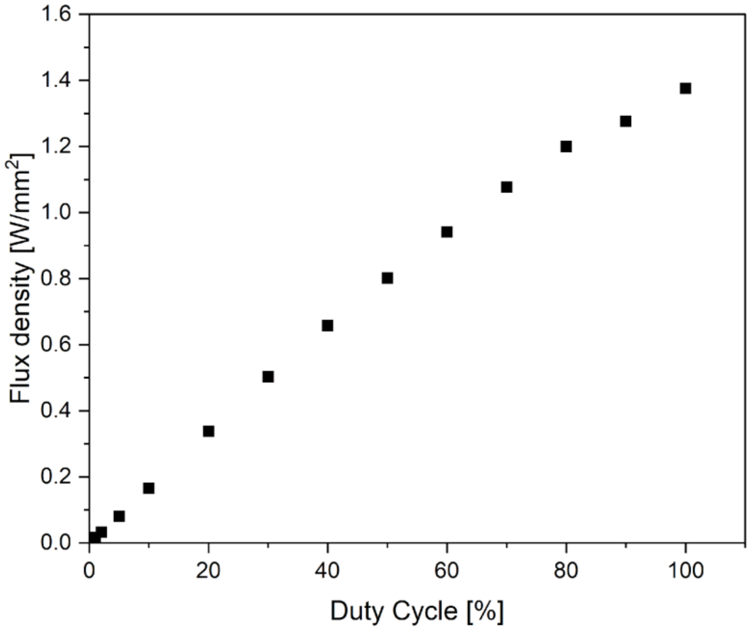


*Fig. S1 Laser power density projected by a 5× objective as a function of laser duty cycle. The illuminated pattern was a rectangle spanning the size of the DMD.*

The grayscale calibration (**Fig. S2**) was required to convert DMD display brightness into physically relevant power values for numerical photothermal simulations. The simulated patterns were scaled according to the calibrated relative laser power, as shown in **Figures S2** and **S3**.

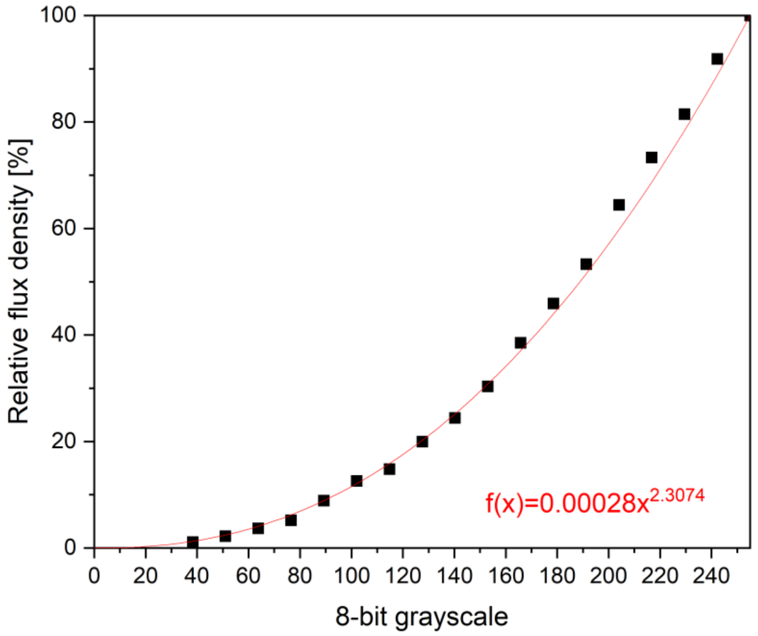


*Fig. S2 Nonlinear dependence of laser power density on the 8-bit grayscale level that controls the DMD on-off duty cycle. The test pattern was a 5 mm diameter circle displayed on the DMD. Measured light power is normalized to the full-white (100%) pattern. The red calibration curve is a least squares fit of the data. The red curve is a least squares fit to log-transformed data.*

The grayscale calibration was necessary to perform numerical simulations of photothermal heating. The pattern used in simulations was scaled to normalized laser power, rather than the display brightness of the pattern, as shown in **Figure S3**.

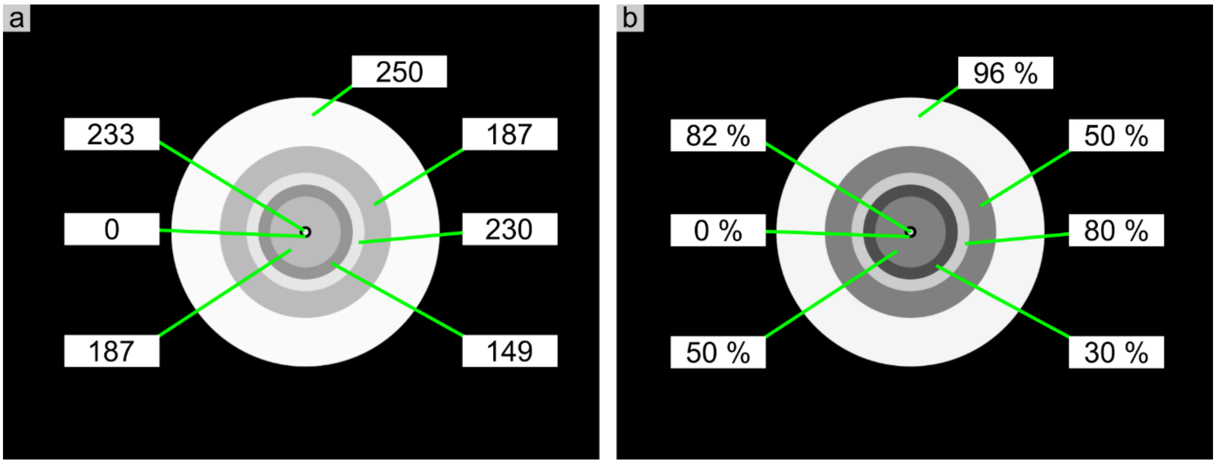


*Fig. S3. Light patterns used to induce top-hat thermal profiles. Comparison of an illumination pattern with intensity mapped as an 8-bit grayscale display brightness (particular to the used projection system) (a) and normalized optical flux values (more universal) (b).*

**1.2** – Substrate and Coating Considerations

Adapting the photothermal platform to other substrates (e.g., Si) is possible but not straightforward. Silicon's much higher thermal conductivity increases heat loss, requiring a stronger laser (our group used a high-power IR source for BCP on Si, DOI:10.1021/acsnano.0c00696), which raises practical issues here because near-IR penetrates Si-based DMDs and an invisible beam is harder to focus. The $Al_2O_3$ coating may be omitted (reducing Ge air stability) or replaced by more robust barriers (e.g., $Si_3N_4$, DOI:10.1038/ncomms8448) or $SiO_2$. Germanium is a durable broadband absorber ($T_m$ = 938 °C) with a visible penetration depth of several tens of nanometers. Low-thermal-conductivity substrates (glass, quartz, fused silica) are preferable for high-resolution photothermal work because they limit lateral heat spread. We have also used intrinsically absorbing bulk and nanomaterials e.g., liquid crystals (DOI:10.1002/adma.202310197), gold nanoparticles (DOI:10.1021/acs.nanolett.5c02830), and some polymers, which produce less surface-localized heating than Ge.

## Note 2 – Block Copolymer Thin Film Preparation and Laser Annealing

**2.1**. BCP Film Thickness Measurements

A separate series of samples on silicon (100) substrates was prepared for X-ray reflectometry measurements. Random brush and BCP films were deposited according to the same procedure as for the photothermal substrates. The film thicknesses are listed in **Table S1** below. These values are also presented in **Figure S4**.

*Tab. S1. Film thickness measurements.*

| Material | Spin coating speed [rpm] | Thickness [nm] | Thickness w/o rand [nm] |
|---|---|---|---|
| **Random brush** | 3000 | 6.07 | - |
| **L45 + rand** | 1500 | 45.7 | 39.6 |
| **L45 + rand** | 2000 | 41.1 | 35.1 |
| **L45 + rand** | 2500 | 37.6 | 31.5 |
| **L45 + rand** | 3000 | 35.9 | 29.8 |
| **L45 + rand** | 4000 | 34.1 | 28.0 |
| **L45 + rand** | 5000 | 30.3 | 24.3 |
| **L45 + rand** | 6000 | 29.4 | 23.3 |
| **L45 + rand** | 8000 | 27.5* | 21.4* |

**The thickness for 8000 rpm was extrapolated using all the thickness measurement data points.*

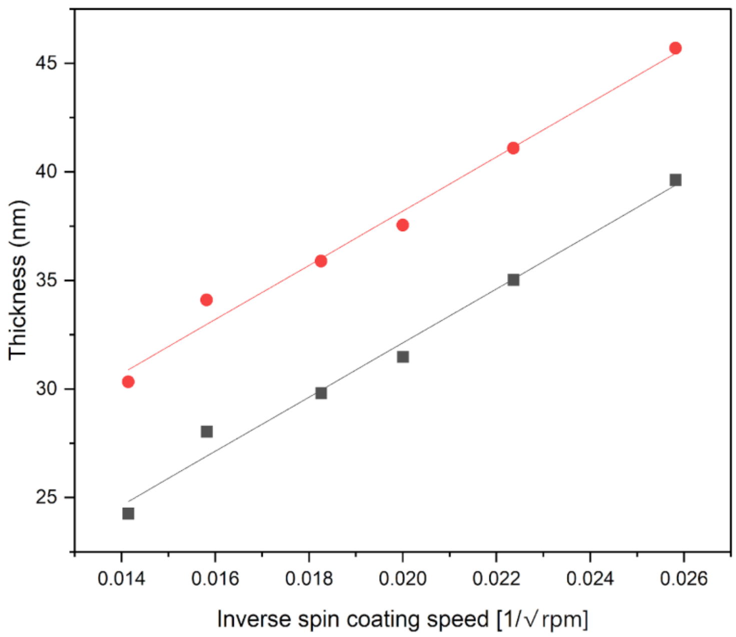


*Fig. S4. Film thickness depends on the reversed, inverted, and squared root spin coating speed. Black symbols: BCP films without the random-copolymer brush; red symbols: BCP films on top of the random-copolymer PS-r-PMMA brush.*

**2.2** Uniformity of BCP Grain-Size within Illuminated Zone

The consistent plateau of grain-size (52 nm ± 2.6 nm) values across the majority (≥ 80%) of the central illuminated region (1 mm in diameter) confirms the high thermal uniformity of the T2O model prediction of the top-hat thermal profile ($T$ = 210 °C, $t$ = 50 s, $d$ = 25 nm). The pronounced decrease in grain size is observed only at the profile's periphery, corresponding to the steep boundary of the top-hat profile demonstrating the effective minimization of thermal gradients within the central zone by the model.

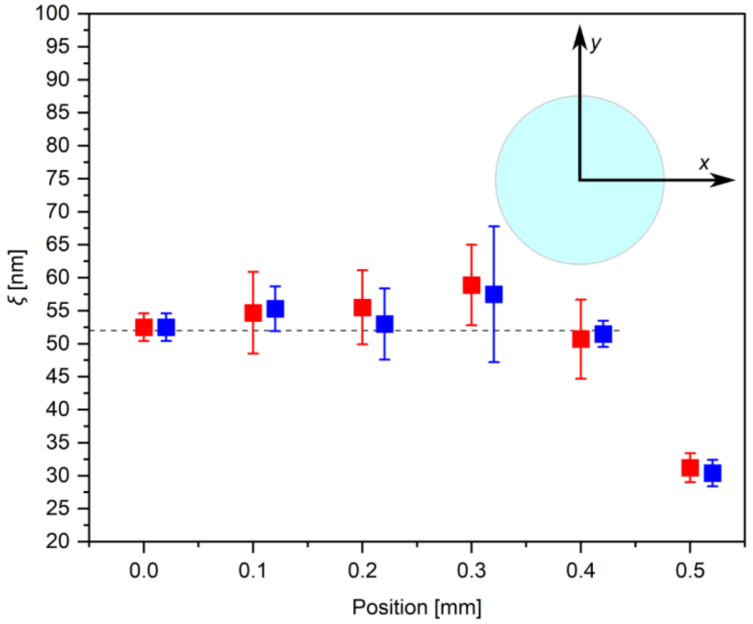


*Fig. S5. Spatial distribution of the BCP grain size as a function of the radial position across the photothermally annealed region (1 mm in diameter). Data points were acquired along two orthogonal axes (x – red symbols and y – blue symbols) mapping the BCP morphology over the illuminated spot, with a spatial step-size of 0.1 mm, starting from the center. The dashed line represents the average value of ξ, calculated by excluding two peripheral points (0.5 mm from the center).*

## 2.3 BCP Ordering Kinetics in Control Hot-Plate Annealing

Laser annealing (LA) outperforms hot-plate annealing (**Figure S6**) for short, high-temperature transients (e.g., 5–10 s), producing larger grains and higher pre-exponential factors A, because LA can access very short thermal pulses that a sample on an aluminum hot plate cannot: we find ≈15 s is required for the sample to reach 90% of the target $\Delta T$ on the hot plate (simulated and experimentally verified). Dewetting occurs with both methods, but significant dewetting appears at much longer times for hot-plate annealing (100 s) than for LA (10 s), reflecting the time-lag and offset between the hot-block setpoint (verified with a calibrated TC probe) and the actual film surface temperature. The slower surface heating and additional oxidative degradation during hot-plate annealing likely explain the near-arrested grain growth observed at nominal block temperatures of 160 °C and 210 °C.

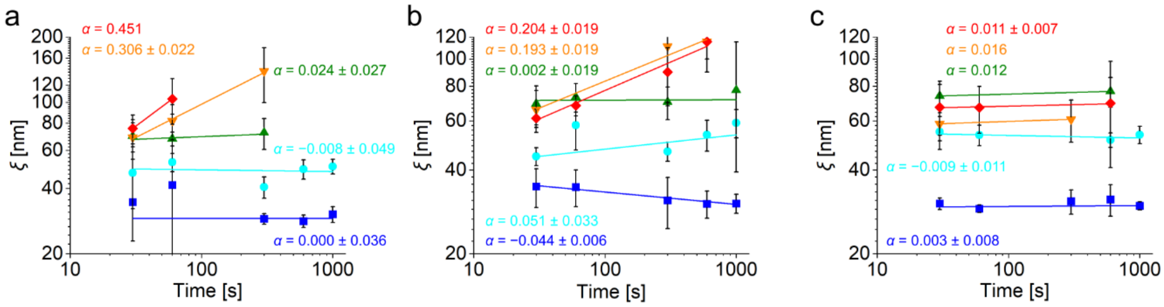


*Fig. S6. Correlation length, ξ versus time in hot-plate annealing of L45-SMMA films with thickness 21 nm (a), 25 nm (b), and 28 nm (c). Solid lines are the best fits to the kinetic power-law model. Hot-plate block temperatures: 160 °C (blue), 210 °C (cyan), 250 °C (olive), 290 °C (orange), 310 °C (red).*

## 2.4 Thermal Degradation and Dewetting of BCP Films

High temperatures during processing can degrade the polymer and, in extreme cases, the underlying glass and germanium layers. Thermal damage is visible to the naked eye after severe exposure to the laser beam and can easily be detected by SEM. Dewetted regions are readily identified by a bluish hue, indicating film discontinuities. In some samples, the extent of dewetting was minor and permitted grain-size analysis, but these conditions were excluded from the kinetic study because they lack practical relevance. Notably, the laser-treated area remains visible after prolonged exposure even when samples appear undamaged and non-dewetted.

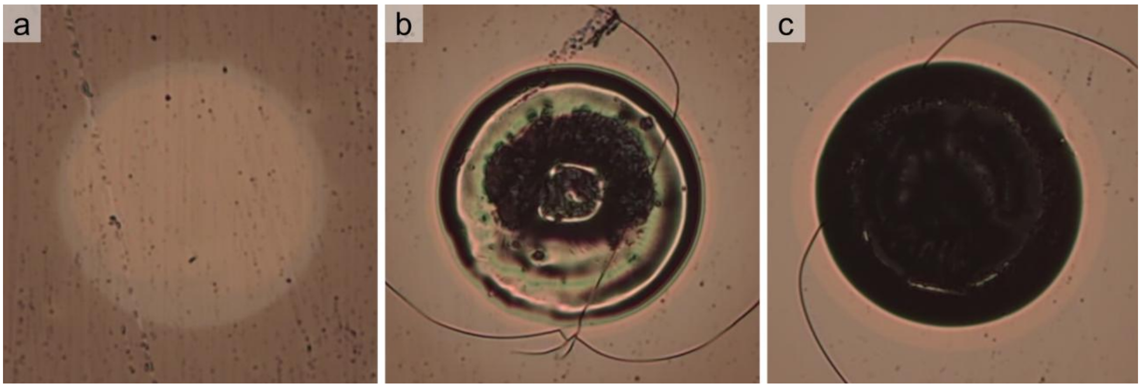


*Fig. S7. Optical micrographs of illuminated samples: (a) thermally undamaged, (b) damaged, and (c) severely damaged. Hairline cracks in (c) indicate glass substrate fracture.*

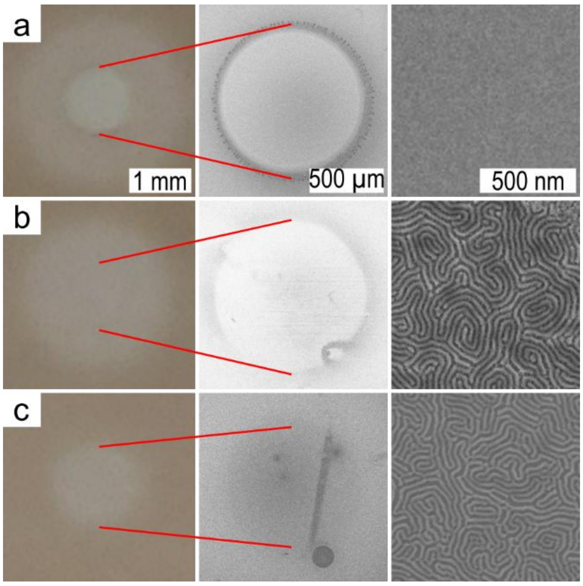


*Fig. S8. Typical appearance of L45-SMMA BCP films after laser annealing, revealed by optical microscopy and SEM. (a) High-power illumination ($T_{max}$ > 350 °C) causes central dewetting with a characteristic ring of material at the illuminated zone perimeter. (b) Lamellar BCP morphology at intermediate temperatures (≈200–250 °C). (c) Finer-grained morphology for T < 200 °C. Samples were converted to $Al_2O_3$ replicas prior to imaging.*

**2.5** BCP conversion to $Al_2O_3$, BCP ashing and Metal Oxide Nanowire Sintering

When the objective is changed from mag 5× to 10×, the temperature difference rises approximately twofold, reaching a maximum of 350 °C. Additionally, the temperature of a heated bed, 130 °C, was used to achieve the maximum temperature of 480 °C. Laser-processed alumina replica of PMMA lamellae remains unaffected, as seen by comparing panels a and b of **Figure S9**. However, during prolonged exposure to temperatures exceeding 400 °C, we noted glass substrate damage.

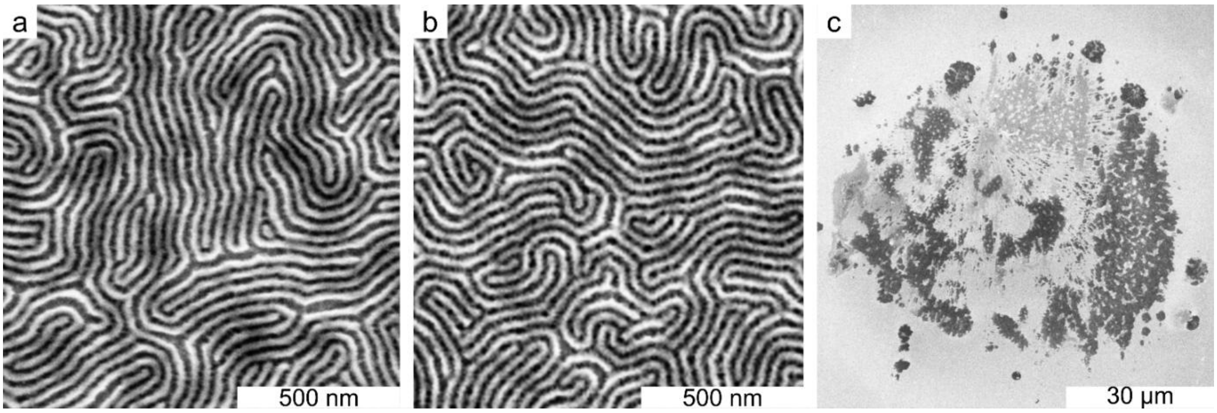


*Fig. S9. Comparison of the SEM images of $Al_2O_3$ replica before sintering (a) and after sintering (b) for 10 s at 400 °C. Exemplary thermal damage after sintering in oxygen atmosphere for 10 s at 480 °C.*

SEM images of the sintered nanowires processed without additional oxygen flow in the ambient laboratory atmosphere are shown in **Figure S10**. Quantitative data of NWs diameter and carbon content vs. sintering temperature are shown in **Figure S11**.

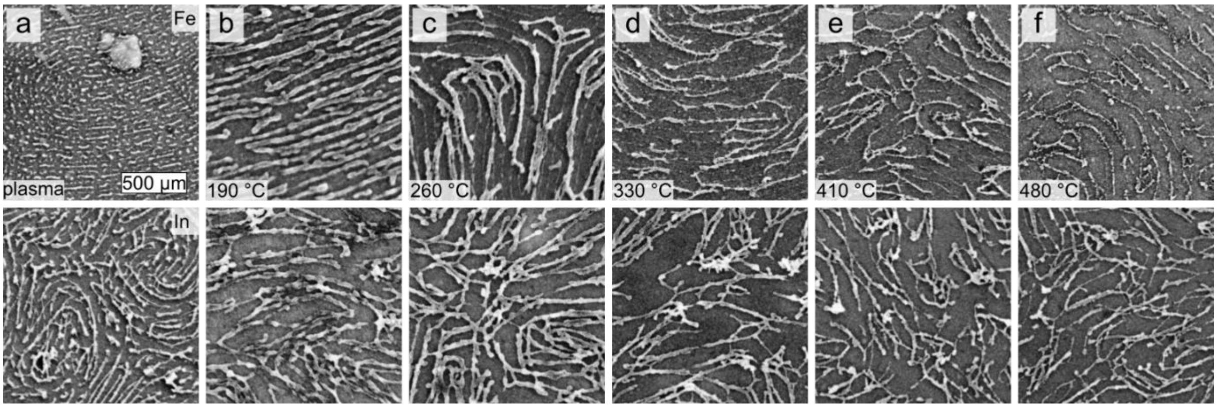


*Fig. S10. SEM images of $Fe_2O_3$ and $In_2O_3$ nanowires processed with oxygen plasma (a) or photothermally heated to the given temperatures (b–f). Laser heating was done in an air atmosphere.*

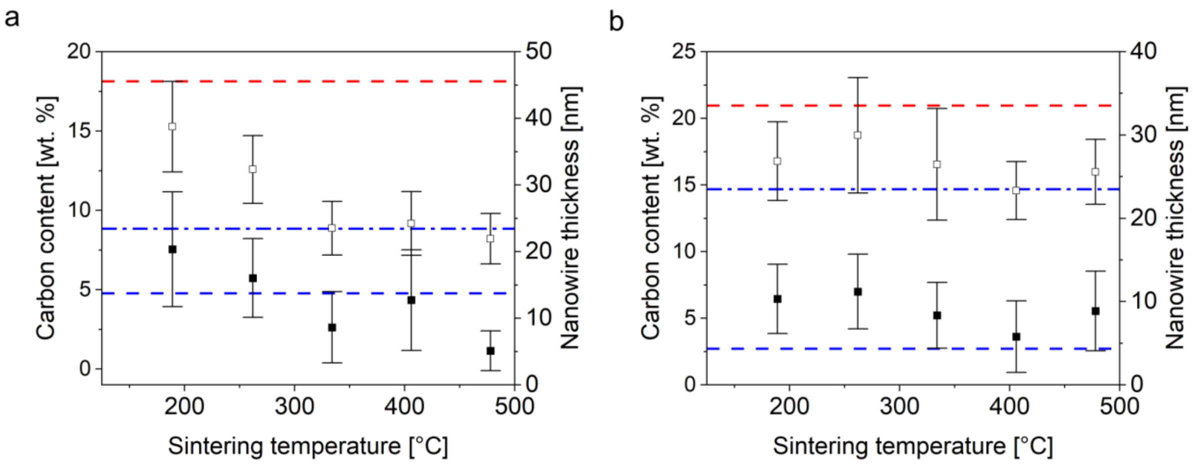


*Fig. S11 Residual carbon content in $Fe_2O_3$ (a) and $In_2O_3$ (b) nanowires after ashing in air (solid symbols). Red dashed line: carbon content of the unprocessed polymer infused with metal acetylacetonates. Blue dashed line: carbon content after oxygen-plasma removal of the BCP template (reference). Open symbols: nanowire thickness following photothermal sintering. Blue dash-dot line: wire thickness after standard oxygen-plasma treatment. The error bars in carbon content represent the standard deviation based on at least five individual EDS measurements, while the error bars for nanowire diameter indicate the standard deviation from 50 individual measurements.*

## Note 3. Thermography and Numerical Simulations of Photothermal Heating

### **3.1.** Thermography

Thermography was performed with an Optris Xi400 thermal camera fitted with an additional germanium objective. The camera's native resolution is 382 × 288 pixels. Measurements used two configurations: (1) the experimental setup with the camera angled above the sample, and (2) a transmission setup in which the heater was replaced by ITO-coated glass (ITO side up), the sample was mounted on the glass with the germanium side down, facing the camera, and the camera was positioned below the sample along the laser axis (**Fig. S12**). A gold finger-patterned glass calibration sample established the spatial calibration: 96.5 px $mm^{-1}$ (≈ 10.4 µm per pixel). According to the manufacturer, reliable temperature readings require an area span of at least 3 × 3 pixels.

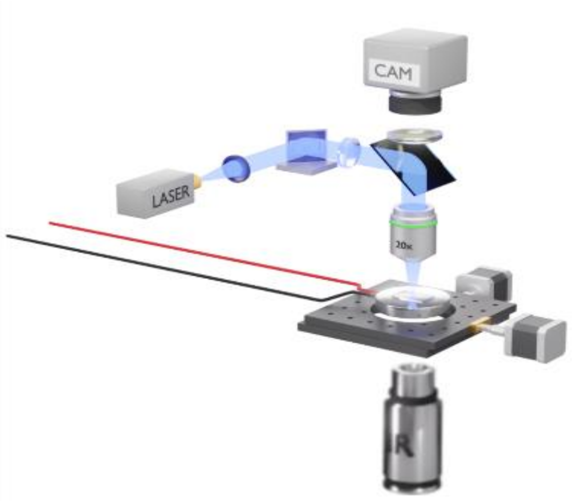


*Fig. S12. Schematic view of IR camera measurements in the transmission configuration.*

The transmission geometry yields non-distorted images (**Fig. S13**) but it has certain drawbacks; the laser beam must pass through the ITO layer, glass, immersion oil, the sample, and their interfaces, causing partial reflection and absorption by ITO. This distributed absorption produces a temperature rise that effectively heats the sample from roughly 1 mm away, slightly blurring and biasing the recorded thermal map.

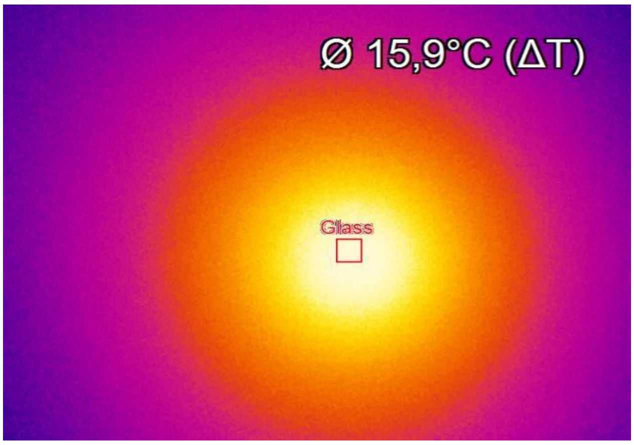


*Fig. S13. The thermal image of the ITO heater illuminated with a top-hat optical pattern. Substrate base temperature, 100 °C, was set before illumination and was subtracted from this thermogram.*

**3.2.** Numerical simulations

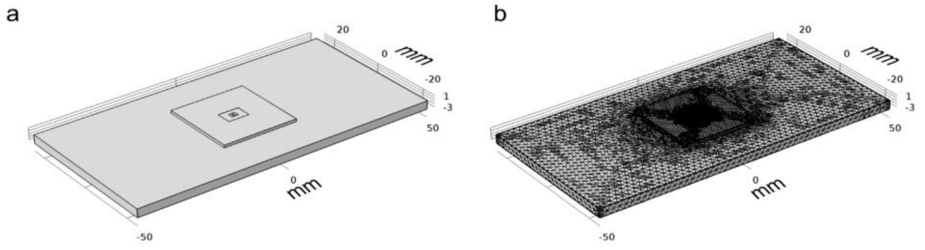


*Fig. S14. Geometric model used for finite-element numerical simulation.*

The bottom part of the model was an aluminum plate representing the resistively-heated base plate (**Fig. S14**). The illuminated region was meshed very finely (maximum element size 10 µm), with a surrounding fine mesh rectangle at 2 mm to avoid discontinuities in the thermal field. A higher-density mesh in the sample center provided improved local accuracy with moderate computation time. The plate base was held at 100 °C (1$^{st}$ type BC). The sample rested on the plate separated by a thermal gap with conductance $h_g$ = 100 W·m$^{-2}$·K$^{-1}$. Patterned light field simulated as a structured surface power source was applied to the sample top layer according to the grayscale projected image. All external faces experienced convective heat flux with $h$ = 20 W·m$^{-2}$·K$^{-1}$ and ambient temperature $T_{ext}$ = 25 °C. In the physical setup, a plexiglass cover limited natural airflow and allowed the local air temperature near the sample to rise slightly; this modest change in air cooling has only a minor effect on temperature distribution in the illuminated area.

*Tab. S2. Thermophysical properties of materials and parameters used in simulations*

| **Material** | **Heat capacity at constant pressure [J kg$^{-1}$ K$^{-1}$]** | **Thermal conductivity [W m$^{-1}$ K$^{-1}$]** |
|---|---|---|
| Aluminum | 904 | 237 |
| Schott BK7 glass | 0.86 | 1.11 |
| **Other parameters** | | |
| Heat transfer coefficient | $h$ | 20 [W·m$^{-2}$·K$^{-1}$] |
| Thermal gap conductance | $h_g$ | 100 [W·m$^{-2}$·K$^{-1}$] |

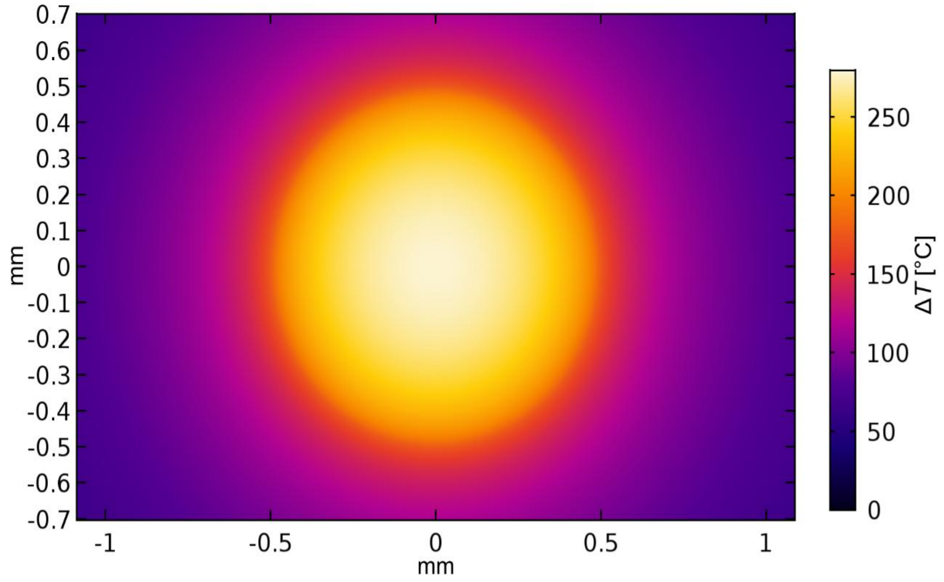


*Fig. S15. An exemplary result of the numerical simulation of laser illumination.*

To account for absorption and reflection, we introduced an effective absorption coefficient *k*. Its value was calibrated by comparison with thermographic measurements. The simulation was then used to generate top-hat illumination patterns and to estimate process temperatures for the 10× objective, where the IR camera resolution was insufficient for direct measurement

## Note 4. Machine Learning in O2T and T2O Models

To predict the required optical profile for achieving a desired thermal field, two models were trained using Keras/TensorFlow software in a Python environment. For this study, only concentric optical fields consisting of 8 annular regions (rings) (each with increasing diameter) were considered. The choice of eight regions was a compromise between maintaining model simplicity for efficient training and ensuring sufficient flexibility to generate complex thermal profiles. To generate the training data, 543 randomly generated optical profiles were projected onto a sample surface, and the resulting thermal fields were recorded using the thermal camera. These measurements were then converted into optical profile–thermal profile pairs by averaging the optical flux power and the corresponding increase of local temperature above the base temperature of the substrate ($\Delta T$) as a function of the distance from the center. This dataset was used to train the first model, referred to as the O2T model (**Fig. S16a**), which takes an optical profile as input and predicts the thermal profile as output. The architecture of the O2T model consisted of three segments, each containing two 1D convolution layers followed by a 1D max pooling layer. The output was then flattened and passed through two dense layers, shaped to match the number of points in the thermal profile. For training, a custom loss function was developed to separately evaluate the thermal drift (i.e., the difference between the means of the real and predicted thermal profiles, denoted as $\Delta T_{\text{drift}}$) and the shape difference (SMSE). SMSE was calculated by adjusting for $\Delta T_{\text{drift}}$ by adding it to the predicted profile and then computing the mean squared error between the real profile. These components were combined with specific weights to obtain the final loss value:

$$loss = a \cdot \Delta T_{\text{drift}} + b \cdot SMSE$$

The weights were tuned to achieve optimal results, with the final values set as $a = 0.5$ and $b = 1$. This custom loss function was used to account for slight variations in thermal drift between measurements due to environmental factors. **Figure S16b** and **Figure S16c** show two examples of model predictions, showing the input optical profile, the experimentally obtained thermal profile, and the predicted thermal profile. The trained O2T model was then used to train a second model, denoted as the T2O model, which predicts the optical profile required to achieve a specified thermal profile. During training, random thermal profiles were generated and given to the model as input, which then predicted the required optical profile. It was then fed to the model O2T that predicted the resulting thermal profile, eliminating the need to experimentally collect thermal profiles during training. The loss was then calculated between the resulting thermal profile and the desired profile given to the model T2O using the same method described for the O2T model. The training scheme for the T2O model is illustrated in **Figure S16d**, with examples of its performance shown in **Figure S16e** and **Figure S16f**. The model was trained until the loss value reached a stable minimum.

Both ML models are reliable only for systems homologous to their training data, i.e., substrates with similar thermal properties. Differences in photon thermalization (and thus heating) due to absorber-layer thickness can be accounted for by linearly scaling $\Delta T$, provided the absorber is much thinner than the substrate.

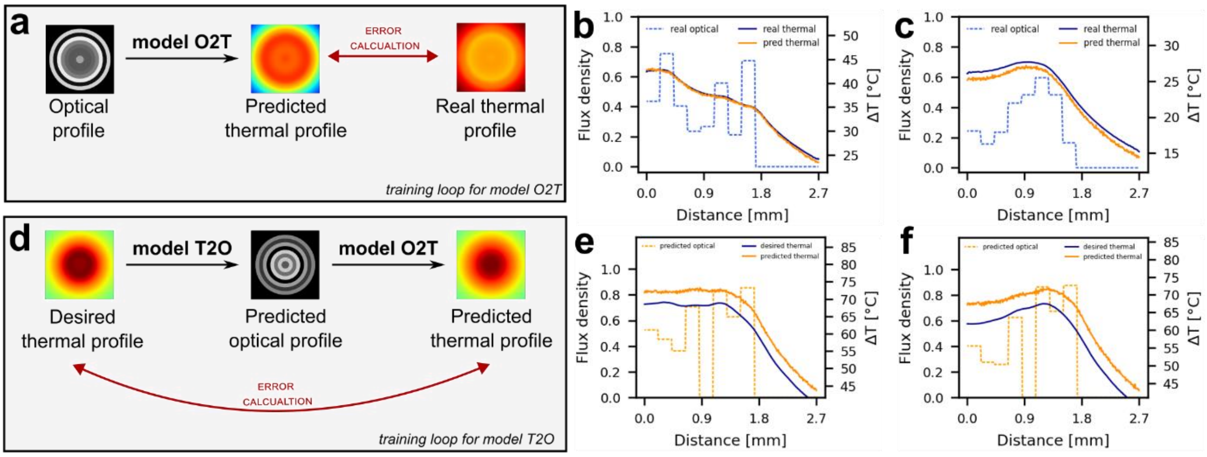


*Fig. S16. Training of the models O2T and T2O. Panel a shows the training scheme for the model O2T: thermal profile is taken as input, the model produces the predicted thermal field, and the loss is calculated by comparing it to the real thermal profile experimentally obtained. Panels b and c show the results of the O2T model: the blue dotted line is the real optical profile, along with the resulting experimentally-collected thermal profile. The orange line shows the thermal profile predicted by the model. The flux is given as a grayscale on the optical fields projected onto the sample (values in the range 0–1). Panel d shows the training scheme of the model T2O: the model is given the desired thermal profile (randomly-generated during training) as input and predicts the optical profile, which is then fed to the model O2T to get the expected thermal profile. Comparing the desired thermal profile with the expected is used to calculate the loss function. Panels e and f show the result of the training of the model T2O: the blue solid line shows the desired thermal profile; the orange dotted line shows the optical profile predicted by the model T2O, and the solid orange line shows the thermal profile obtained by feeding the predicted optical profile to model O2T.*
